# Isolated Cranial Nerve-III Palsy Secondary to Perimesencephalic Subarachnoid Hemorrhage

**DOI:** 10.1155/2016/6319548

**Published:** 2016-02-01

**Authors:** Justin R. Abbatemarco, Hussam A. Yacoub

**Affiliations:** ^1^The Neurology Institute, Cleveland Clinic, 9500 Euclid Avenue, Cleveland, OH 44195, USA; ^2^Division of Neurology, Center for Advanced Health Care, Lehigh Valley Health Network, 1250 South Cedar Crest Boulevard, Allentown, PA 18103, USA

## Abstract

We describe isolated cranial nerve-III palsy as a rare clinical finding in a patient with perimesencephalic subarachnoid hemorrhage. In this unusual case, the patient presented with complete cranial nerve-III palsy including ptosis and pupillary involvement. Initial studies revealed subarachnoid hemorrhage in the perimesencephalic, prepontine, and interpeduncular cisterns. Angiographic studies were negative for an intracranial aneurysm. The patient's neurological deficits improved with no residual deficits on follow-up several months after initial presentation. Our case report supports the notion that patients with perimesencephalic subarachnoid hemorrhage have an excellent prognosis. Our report further adds a case of isolated cranial nerve-III palsy as a rare initial presentation of this type of bleeding, adding to the limited body of the literature.

## 1. Introduction

Spontaneous subarachnoid hemorrhage (SAH) can be caused by a cerebral aneurysm rupture. In approximately 15% of patients with SAH, the source of bleeding cannot be identified on repeated catheter or computed tomography angiographic studies [1, 2]. Patients with nonaneurysmal SAH with CT findings of blood limited to the perimesencephalic cisterns were first described by van Gijn and colleagues [3]. The bleeding pattern in cases of angiographic-negative SAH can be divided into two groups. In the first group, bleeding is confined to the midbrain cisterns with no evidence of intraventricular extension, a pattern described as perimesencephalic or prepontine. In the second group of angiogram-negative SAH, bleeding follows an aneurysmal pattern, involving the Sylvian and interhemispheric fissures. Patients in the first group, with perimesencephalic nonaneurysmal subarachnoid hemorrhage (PNSH), have an excellent prognosis [4, 5] and a lower risk of bleeding recurrence.

Herein, we describe a case of rarely reported isolated complete cranial nerve-III (CN-III) palsy secondary to PNSH.

## 2. Case Description

**Figure 1:**
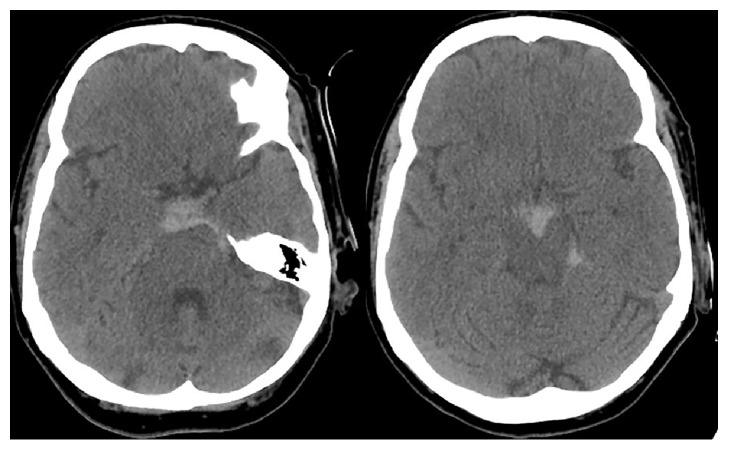
CT of the head demonstrated subarachnoid hemorrhage in the perimesencephalic, prepontine, interpeduncular cisterns.

**Figure 2:**
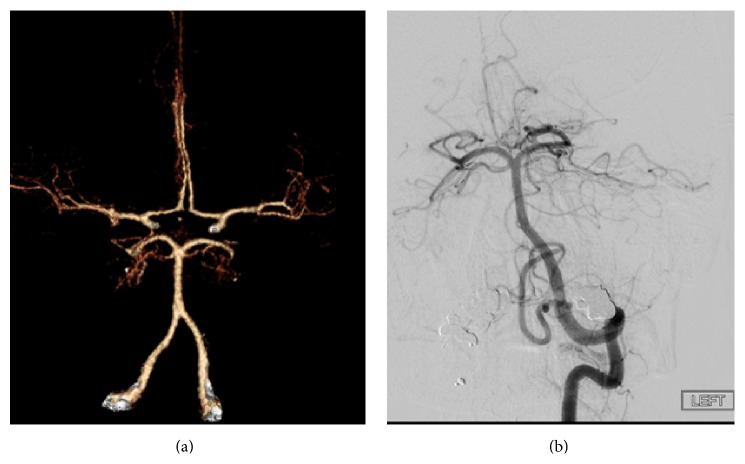
CT angiography (a) and a 4-vessel cerebral arteriogram (b) showed no intracranial aneurysms.

A right-handed 63-year-old woman with a past medical history of essential hypertension and hyperlipidemia presented with severe headache and difficulty opening her right eye. She denied ocular pain. She was on warfarin for portal vein thrombosis, with an international normalized ratio (INR) of 2.5. Initial neurological evaluation revealed right-sided severe ptosis, 8 mm nonreactive pupil, and limited adduction and vertical gaze. Severity on the Hunt and Hess scale was assessed as grade II. A computed tomography (CT) of the head showed SAH in the perimesencephalic, prepontine, and interpeduncular cisterns (Figure 1). Angiographic studies were negative for an intracranial aneurysm (Figure 2).

Fresh frozen plasma was immediately administered; repeat INR was 1.4. The patient was admitted to the neuroscience intensive care unit and a follow-up CT revealed a stable SAH. The CN-III palsy gradually improved and was completely resolved 3 days after admission. Magnetic resonance imaging of the brain revealed no brain stem ischemia. The patient was discharged home in a stable condition with no recurrent neurological symptoms. Follow-up evaluation several weeks after discharge revealed no recurrent symptoms and no deficits on neurological examination. The patient declined recommended follow-up imaging studies.

## 3. Discussion

Defined by hemorrhage into the brain stem and suprasellar cisterns without diffusion into the Sylvian fissure or lateral ventricles [6], PNSH contributes to between 8% and 11% of nontraumatic SAH cases but comprises from 21% to 68% of angiogram-negative cases [7]. Patients with PNSH typically present with a sudden onset of excruciating headache, usually with a Hunt and Hess grade I or grade II on initial presentation [7]. Focal neurological findings are very rare. One study reported only 10 of 127 patients with PNSH to have focal neurological deficits including hemiparesis, leg paresis, and facial and abducens nerve palsies [8]. Isolated complete, pupillary-involving CN-III palsy has been rarely reported as a complication of PNSH [9, 10].

The exact underlying pathophysiology of PNSH is still not well understood. Potential sources of PNSH include small arteriovenous malformation, arterial or venous bleeding secondary to thrombosis, or capillary bleeding [6]. Potential causes of complete CN-III palsy in a patient with PNSH include mass effect secondary to hematoma, toxic degradation of blood products, and brain stem ischemia [9]. Vasospasm is a rare complication of nonaneurysmal SAH; however, it was mentioned in one study in which magnetic resonance angiography revealed a spastic basilar artery [9]. In our patient, MRI of the brain on initial presentation did not reveal any brain stem ischemia, but this does not inevitably exclude small vessel insufficiency due to vasospasm or mass effect. The MRI establishes solely that permanent infarction of tissue has not yet occurred. Other potential causes of CN-III palsy in a patient with PNSH include a transient elevation in intracranial pressure mediating venous congestion, eventually causing hypoperfusion of the oculomotor nerve. The lack of ocular pain in our patient further supports nerve ischemia as a potential mechanism, as painful lesions are more commonly associated with a compressive pathology.

Compared to a reported good prognosis for 64% of patients with aneurysmal SAH, patients with PNSH had 100% favorable outcomes at 8-month follow-up in a 1993 retrospective study [11]. More recently, prognostic factors and clinical outcomes of nonaneurysmal SAH in 125 patients from a single center were analyzed prospectively, with favorable outcomes reported in 83% overall and in 88% of the patients with PNSH. Overall, good outcomes were associated with younger age, good admission status, and the absence of hydrocephalus [12]. In another recent study, Cánovas et al. [13] retrospectively evaluated outcomes in 108 patients with nonaneurysmal SAH over a mean follow-up period of 5.5 years and found that patients with PNSH had benign courses and excellent short- and long-term prognoses. Furthermore, patients with an initial grade of III or IV on the Hunt and Hess scale due to hydrocephalus and vasospasms were found to have more complications and worse outcomes. Our case report supports the overall notion of excellent prognosis in patients with PNSH as demonstrated with the rapid recovery of complete CN-III palsy.

The first case of transient facial nerve palsy was recently reported in a patient with PNSH [14]. The case illustrated that, in addition to the classic clinical symptoms of headache, nausea, and vomiting, a cranial neuropathy may also occur. The case also supports the good prognosis of neurological deficits, such as cranial neuropathies, associated with PNSH.

In summary, we report a case of isolated complete CN-III palsy as a rare complication of PNSH. Our case also provides evidence that an intracranial aneurysm is not the solitary mechanism to consider in a patient with a sudden onset of complete CN-III palsy and supports the good prognosis previously reported in the small body of literature.
